# Effectiveness and cost-effectiveness of a group-based pain self-management intervention for patients undergoing total hip replacement: feasibility study for a randomized controlled trial

**DOI:** 10.1186/1745-6215-15-176

**Published:** 2014-05-20

**Authors:** Vikki Wylde, Elsa Marques, Neil Artz, Ashley Blom, Rachael Gooberman-Hill

**Affiliations:** 1 Musculoskeletal Research Unit, University of Bristol, Learning and Research Building, Southmead Hospital, Bristol BS10 5NB, UK; 2 School of Social and Community Medicine, University of Bristol, Canynge Hall, 39 Whatley Road, Bristol BS8 2PS, UK

**Keywords:** Feasibility, Group intervention, Pain self-management, Randomized controlled trial, Total hip replacement

## Abstract

**Background:**

Total hip replacement (THR) is a common elective surgical procedure and can be effective for reducing chronic pain. However, waiting times can be considerable. A pain self-management intervention may provide patients with skills to more effectively manage their pain and its impact during their wait for surgery. This study aimed to evaluate the feasibility of conducting a randomized controlled trial to assess the effectiveness and cost-effectiveness of a group-based pain self-management course for patients undergoing THR.

**Methods:**

Patients listed for a THR at one orthopedic center were posted a study invitation pack. Participants were randomized to attend a pain self-management course plus standard care or standard care only. The lay-led course was delivered by Arthritis Care and consisted of two half-day sessions prior to surgery and one full-day session after surgery. Participants provided outcome and resource-use data using a diary and postal questionnaires prior to surgery and one month, three months and six months after surgery. Brief telephone interviews were conducted with non-participants to explore barriers to participation.

**Results:**

Invitations were sent to 385 eligible patients and 88 patients (23%) consented to participate. Interviews with 57 non-participants revealed the most common reasons for non-participation were views about the course and transport difficulties. Of the 43 patients randomized to the intervention group, 28 attended the pre-operative pain self-management sessions and 11 attended the post-operative sessions. Participant satisfaction with the course was high, and feedback highlighted that patients enjoyed the group format. Retention of participants was acceptable (83% of recruited patients completed follow-up) and questionnaire return rates were high (72% to 93%), with the exception of the pre-operative resource-use diary (35% return rate). Resource-use completion rates allowed for an economic evaluation from the health and social care payer perspective.

**Conclusions:**

This study highlights the importance of feasibility work prior to a randomized controlled trial to assess recruitment methods and rates, barriers to participation, logistics of scheduling group-based interventions, acceptability of the intervention and piloting resource use questionnaires to improve data available for economic evaluations. This information is of value to researchers and funders in the design and commissioning of future research.

**Trial registration:**

Current Controlled Trials ISRCTN52305381.

## Background

Primary total hip replacement (THR) is one of the most commonly performed elective surgical procedures in the UK, with 76,448 operations recorded in the National Joint Registry for England and Wales in 2012 [1]. The operation is often successful at providing pain relief, most commonly caused by osteoarthritis; however, approximately 10% of patients experience chronic pain in their replaced hip [2]. Patients often wait months or even years for THR surgery despite targets aimed at reducing National Health Service (NHS) waiting times [3]. In this lead up to surgery, patients report high levels of intrusive pain impacting on their lives, lack of information about managing pain, and uncertainty about where to seek advice or support [4,5].

Interventions to support patients with self-management of arthritis can improve pain, self-efficacy, symptom management and psychological well-being [6-10]. Trials of these interventions with patients waiting for joint replacement report positive beneficial effects on pain and skills acquisition [11,12] but the effectiveness and cost-effectiveness of a pain self-management intervention has not yet been evaluated [13]. Prior to conducting such an evaluation it is important to conduct feasibility work, because previous studies of self-management programs for patients with arthritis have faced challenges through low recruitment rates, poor uptake of the intervention and high attrition rates [8,14-17].

Feasibility and pilot work to explore trial processes can include testing trial procedures and data collection methods, randomization processes, recruitment rates, and attrition rates [18,19]. This preliminary work can often highlight unanticipated issues with trial design and conduct [20,21], which can then be addressed to maximize the success of intervention evaluation in a full-scale randomized controlled trial (RCT). This can increase the efficiency of research funding by evaluating the likely success of processes before undertaking a definitive trial. The importance of feasibility work to evaluate trial processes has been highlighted in a systematic review of cluster RCTs in primary care, which concluded that a number of reported issues with recruitment, adherence to trial protocol and data collection methods could have been pre-emptively identified and addressed through feasibility work [22]. In addition to testing trial processes, another objective of preliminary work prior to a full-scale RCT can be to test the acceptability of an intervention, particularly if the intervention is complex in nature [23]. Preliminary work to develop, refine and pilot complex interventions is recommended by the Medical Research Council [24]. Early evaluation of the acceptability of a complex intervention can highlight aspects of the intervention that can then be modified prior to a definitive trial [25-27].

The aims of this study were two-fold: first to evaluate the feasibility of conducting an RCT to assess the effectiveness and cost-effectiveness of a group-based pain self-management course for patients undergoing THR, and second to assess the acceptability of the intervention. Specific objectives were to assess the feasibility of trial design and procedures, ascertain recruitment and retention rates, identify barriers to participation, develop resource-use data collection methods, assess questionnaire completion rates, and evaluate uptake and patient satisfaction with the course.

## Methods

### Design and ethics

The study was a single-center feasibility study of an RCT. The study was approved by the South West Central Bristol Research Ethics Committee (reference 11/SW/0056) and all participants provided their informed, written consent to participate. The trial was registered on the National Institute for Health Research Clinical Research Network Portfolio (UKCRN ID 11270) and ISRCTN register (ISRCTN52305381) on 28 June 2013. A CONSORT checklist for the reporting of this study can be found in Additional file 1.

### Participant recruitment

Between June 2011 and June 2012, patients listed for THR surgery at one elective orthopedic center were posted a study invitation pack. The patient information booklet was designed in collaboration with a patient and public involvement group [28]. Patients interested in participating returned a signed consent form and reply slip to the research team. The inclusion criterion was being listed for a primary THR because of osteoarthritis. Exclusion criteria comprised lack of capacity or unwillingness to provide informed consent, or inability to complete English language questionnaires. To explore whether patients enrolled in the study were representative of those undergoing THR, age and gender of all eligible patients was recorded.

### Telephone interviews with non-participants

Brief telephone interviews were conducted with patients who declined to participate in the study but gave permission to be contacted by a researcher to discuss non-participation. Reasons for non-participation were recorded by the researcher in notes on a standardized form.

### Randomization

Participants were allocated to the intervention or standard care group on a 1:1 ratio using a computer-generated randomization system (Minim) [29]. Allocation was minimized by age and gender to ensure equal distribution between groups. Participants were allocated to treatment group after recruitment. Blinding of researchers and patients was not possible because the intervention involved attending a course. Participants were informed of the results of randomization via letter, and those randomized to the intervention group were telephoned to discuss course arrangements.

### Assessment

Participants completed postal questionnaires at baseline (after recruitment), before surgery, and one month, three months and six months after surgery. If no reply was received after two weeks, a single reminder was sent. The questionnaires included the Western Ontario and McMaster Universities Osteoarthritis Index [30], Pain Self-Efficacy questionnaire [31], Brief COPE [32], Beliefs about Medicines Questionnaire [33], EQ-5D [34] and Functional Co-morbidity Index [35]. Patients also completed questions about socioeconomic status, pain in other joints, fatigue, pain distress, activity levels and pain medication usage. As this was a feasibility study, the results of these questionnaires are not the focus of this article.

### Resource use

Economic evaluations alongside RCTs are increasingly pertinent to evaluate the cost-effectiveness of a novel intervention in a context of scarce NHS resources [36]. The three-month and six-month post-operative questionnaire included a full resource-use questionnaire to identify and measure NHS resources used including community-based doctor and nurse visits, physiotherapy and occupational therapy visits, secondary care inpatient and outpatient visits and medication, use of social services, patient expenses, informal care, and productivity losses incurred in the period. Participants were given a pre-operative resource-use diary to record any resources used from randomization until surgery. They were asked to return the completed pre-operative diary with their one-month post-operative questionnaire. One month and three months post-operation, patients were given a resource-use log to prospectively record their use of resources in the following period in order to aid them in the completion of the resource use questions in the three-month and six-month questionnaires [37]. The aim of these questionnaires was not to formally evaluate the differences in costs and consequences of delivering the intervention, but to refine resource-use data collection methods. Therefore, analyses focused on rates of missing data, which is a common issue with resource-use questionnaires [37].

### Intervention

The Challenging Pain and Keep Challenging Pain courses were delivered by two lay trainers from Arthritis Care, a registered UK charity that has been delivering self-management courses since 1994 [38]. The courses were held at the hospital from which participants were recruited. Reimbursement of travel costs (mileage and parking fees) or a pre-paid taxi was offered to all participants who attended the courses.

#### Challenging Pain course

The pre-operative Challenging Pain course consisted of two sessions running over consecutive weeks, with each session lasting two and a half hours [39]. The emphasis of the course was on pain management and introduced a variety of cognitive pain management techniques, with the aim of providing coping skills to enable patients to manage their pain and its impact more effectively. Delivery involved a combination of presentations, group work, pair work, demonstrations and practical sessions. The first session included introductions to conscious breathing, full body relaxation, exercise, goal setting and managing stress. The second session reviewed these topics and introduced pacing, medications and other therapies, guided imagery, managing negative thoughts, and effective communication.

#### Post-operative Keep Challenging Pain course

The five-hour Keep Challenging Pain course was designed by Arthritis Care, in conjunction with a physiotherapist, to be delivered specifically to post-operative THR patients. The course reviewed pain management strategies introduced in the Challenging Pain course, provided advice on recovery after THR, reviewed goal setting and problem solving, and included a practical exercise session led by a registered physiotherapist.

#### Course evaluation

A short structured feedback questionnaire about the course was completed by participants at the end of the both the Challenging Pain and Keep Challenging Pain courses.

### Sample size

No formal sample size calculation can be performed for a feasibility study. The average sample size for feasibility studies assessing trial design and the acceptability of interventions is around 60 patients [40]. A minimum of 80 patients (40 per arm) was deemed an appropriate sample size for this trial to allow an estimate of recruitment and retention rates and explore the acceptability of the intervention.

### Analysis

In line with recommendations about good practice in the analysis of feasibility studies [18], analysis was descriptive and no comparisons of the outcomes between the two arms of the trial was conducted. Descriptive statistics on recruitment rates, baseline patient characteristics, retention of participants and questionnaire return rates are presented as means and standard deviations (SD) or 95% confidence intervals (CI), medians and interquartile ranges (IQR), or percentages. Resource-use data were considered complete when the patient recorded enough data to allow for costing using a national tariff. Completion rates were reported per question and aggregated per two economic perspectives: the NHS and Personal Social Services (PSS) perspective, and a broader societal perspective. Data on reasons for non-participation were collated and coded into themes by one researcher (VW) and these themes were then discussed and agreed with a second researcher (RGH) [41].

## Results

### Recruitment rate and participants

**Figure 1:**
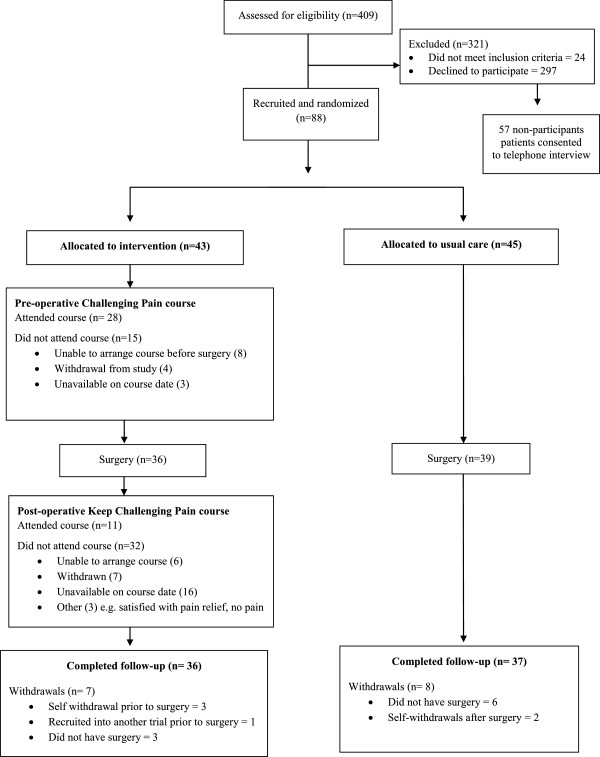
Consort flow diagram.

Postal invitations were sent to 385 eligible patients and 88 consented to participate, giving a recruitment rate of 23% (Figure 1). A total of 297 patients did not return a reply slip and consent form to the research team. Participants’ baseline characteristics are displayed in Table 1. Participants underwent THR surgery at a median of 12 weeks (IQR 8 to 15) after recruitment into the study. Non-participants had a similar median age (67 years, SD 13) to participants but were more likely to be male (46% male).

**Table 1:** Baseline demographic and clinical characteristics of participants

	**Overall**	**Allocated to intervention**	**Allocated to standard care**
	**n = 88**	**n = 43**	**n = 45**
Mean age (SD)	66 (11)	65 (12)	67 (10)
Female: male (%)	65:35	65:35	64:36
Living alone (%)	19	18	20
College or university education (%)	35	32	39
Retired (%)	60	61	59
Mean WOMAC Pain score^a^ (SD)	38 (18)	37 (17)	38 (20)
Mean WOMAC Function score^a^ (SD)	37 (18)	39 (18)	35 (18)
Mean Pain Self-Efficacy score^b^ (SD)	32 (14)	35 (13)	30 (14)

^a^WOMAC Pain and Function scores range from 0 to 100 (worst to best). ^b^Pain Self-Efficacy questionnaire scores range from 0 to 60 (low self-efficacy to high self-efficacy). SD, standard deviation; WOMAC, Western Ontario and McMaster Universities Osteoarthritis Index.

### Reasons for non-participation

Brief telephone interviews were conducted with 57 non-participants (19%). These patients had a mean age of 71 years (SD 10) and 37 (65%) were female. Patients gave 91 reasons for non-participation, most frequently relating to perceptions and views about the pain self-management course (Table 2). These reasons included previously attending pain self-management courses and finding them unhelpful; a perceived lack of need because pain was adequately managed; a dislike of group formats; and concerns over difficulty in attending the course because of pain, age and/or other health conditions. The second most frequently given reason for non-participation concerned issues around traveling to the hospital to attend the course.

**Table 2:** Reasons for non-participation

**Barriers to participation (number of patients)**	**Examples of reasons given**
Thoughts about attending the course (25)	Difficult to sit and concentrate during course because of pain/age/other health conditions
	Dislike of group format
	Found previous pain management course unhelpful
	Can already manage pain
	Course would not be helpful as pain not too bad
	Difficult to attend because of other health conditions
	Would rather spend time doing other things
Difficulty getting to hospital (22)	Unable to drive/use public transport due to hip problems
	Distance to hospital perceived as too far
	Would have to rely on family/friends for transport
	Limited mobility or uses wheelchair
Other commitments (13)	Carer for family member
	Employment
	Children
Questionnaires (8)	Dislike of completing questionnaires
	Difficult to complete because of other health conditions
	Lack of time because of other commitments
Other hospital appointments (6)	Lack of time for additional visits to hospital
	Inconvenient to make additional visits to hospital
Feels already has enough knowledge (6)	Previous hip replacement
	Knows people who have had hip replacement
	Has attended physiotherapy/exercise session
Healthcare-related (6)	Operation may not be going ahead
	Dissatisfied with co-ordination of care
Other (5)	Emigrating
	Recently widowed
	Has taken part in research before

### Retention of participants

Fifteen patients (17%) were withdrawn from the study: seven from the intervention group and eight from the standard care group (Figure 1). In the intervention group, three patients self-withdrew, three patients did not undergo surgery during the study period, and one patient was withdrawn because they were recruited into another trial whose protocol precluded participation in two trials. In the standard care group, two patients self-withdrew and six patients did not undergo surgery during the study period.

### Outcomes assessment and economic evaluation

The questionnaire return rates at each assessment time were high, ranging from 72% to 93% (Table 3). The rate of questionnaire return was similar between trial arms, with less than 10% difference in return rates at each time point, except the three-month post-operative questionnaire, which was returned by more patients in the standard care arm than the intervention arm (91% versus 72%, respectively). Return rates for the pre-operative resource-use diaries were low with only 35% of patients returning their diary.

**Table 3:** Return rates for questionnaires at each assessment time

	**Median (IQR) time of completion**	**Intervention group**	**Usual care group**	**Overall**
**Baseline**	10 weeks (5 to 13) prior to surgery	90% (38/43)	93% (42/45)	91% (80/88)
**Pre-operative**	1 week (0.5 to 1.3) prior to surgery	76% (25/33)	85% (29/34)	81% (54/67)
**Pre-operative resource-use diary**	Pre-operative to one month after surgery	31% (11/36)	39% (15/38)	35% (26/74)
**One month post-operative**	4 weeks (3 to 5) after surgery	89% (32/36)	92% (35/38)	91% (67/74)
**Three months post-operative**	13 weeks (13 to 14) after surgery	72% (26/36)	91% (34/37)	82% (60/73)
**Six months post-operative**	26 weeks (26 to 27) after surgery	89% (32/36)	86% (32/37)	88% (64/73)

IQR, interquartile range.

Table 4 presents the completion rates of resource-use data in the three-month and six-month post-operative questionnaires. For those who returned a questionnaire, completion rates for NHS resource-use questions were high for secondary care resource use (over 90%) and medication use (over 80%), and lower for community-based resources (65% for the intervention arm and 66% for the standard care arm). PSS data also had high completion rates (over 86%), particularly in the intervention group. When accounting for non-returners, completion rates were lower, with community-based resources being the lowest completed category. Overall, data for an economic evaluation from an NHS and PSS perspective were available for 33% of patients in the intervention group and 43% of patients in the standard care group. When considering other categories of resource use beyond health and social care, travel costs was the least completed category. As a result, for an economic evaluation from a societal perspective, complete data were only available for 17% of patients in the intervention group and 19% of patients in the standard care group.

**Table 4:** Completion rates for resource use categories over the follow-up period

**Resource-use category**	**Intervention**			**Standard care**		
	**Number of completes**	**Percentage of returners (n = 26)**	**Percentage of all (n = 36)**	**Number of completes**	**Percentage of returners ** **(n = 29)**	**Percentage of all ** **(n = 37)**
*NHS resource use*						
Community-based visits	17	65%	47%	19	66%	51%
Hospital inpatient visits	24	92%	67%	28	97%	76%
Outpatient and A&E visits	25	96%	69%	28	97%	76%
Prescribed medications	22	85%	61%	24	83%	65%
*PSS resource use*						
Home care worker	26	100%	72%	27	93%	73%
Food at home services	26	100%	72%	26	90%	70%
Social worker visits	26	100%	72%	25	86%	68%
Home changes	24	92%	67%	25	86%	68%
***NHS + PSS perspective***	***12***	***46%***	***33%***	***16***	***55%***	***43%***
*Other resources: productivity losses, informal care, private expenses and other*						
Time off work	23	89%	64%	24	83%	65%
Time off usual and leisure activities	26	100%	72%	21	72%	57%
Informal care time	26	100%	72%	23	79%	62%
Charities and support group visits	26	100%	72%	25	86%	68%
Privately paid therapies used	23	89%	64%	25	86%	68%
Travel costs	13	50%	36%	14	48%	38%
Over-the-counter medications	25	96%	69%	27	93%	73%
***Societal perspective***	***6***	***23%***	***17%***	***7***	***24%***	***19%***

Note: Number of participants completing the questions in a resource-use category at both three- and six-months follow-up. Data are presented for the 73 patients in the trial at final follow-up. A&E, Accident and Emergency; NHS, National Health Service; PSS, Personal Social Services.

### Acceptability of the intervention

#### Pre-operative Challenging Pain course

Four pre-operative Challenging Pain courses were held, with four to nine participants attending each course. Of the 43 participants randomized to the intervention group, 28 attended the pre-operative course (17 attended both sessions, 11 attended one session) at a median of five weeks prior to surgery (IQR 2 to 8). Reasons for non-attendance are presented in Figure 1. Results from the course evaluation questionnaire are presented in Table 5. Free text comments on the evaluation questionnaires frequently gave positive feedback on the group format of the course as this provided the opportunity to meet other people undergoing THR.

**Table 5:** Results from the challenging pain and keep challenging pain evaluation questionnaires

	**Challenging pain course**	**Keep challenging pain course**
	**(n = 27)**	**(n = 11)**
Has the course been useful? (% yes)	100	100
Recommend for other THR patients? (% yes)	100	100
Mean usefulness (95% CI)	7.3 (6.5 to 8.1)	8.9 (8.4 to 9.5)
Mean satisfaction with content (95% CI)	8.0 (7.2 to 8.7)	9.0 (8.4 to 9.6)
Mean satisfaction with delivery (95% CI)	8.4 (7.7 to 9.0)	9.0 (8.2 to 9.8)

Usefulness and satisfaction questions rated on 0 to 10 scale (worst to best). CI, confidence interval.

NB: One patient attended the Challenging Pain course but did not complete an evaluation questionnaire.

#### Post-operative Keep Challenging Pain course

Three post-operative Keep Challenging Pain courses were held but were poorly attended, with two to five participants on each course. The courses were attended by 11 patients at a median of nine weeks post-operative (IQR range 5 to 14). Reasons for non-attendance are presented in Figure 1. Results of the course evaluation questionnaire are presented in Table 5. Free text comments on the evaluation questionnaires most frequently gave positive feedback on the physiotherapy session and the group format of the course.

## Discussion

This study looked at the feasibility of an RCT to evaluate the effectiveness and cost-effectiveness of a group-based pain self-management intervention for patients undergoing THR and the acceptability of this intervention. Although feasibility studies are conducted to address trial design and methodology, a systematic review found that articles often include only a minimal discussion of the methodological findings and implications [42]. This feasibility study highlighted several methodological considerations that warrant further discussion.

### Barriers to participation

It is important to explore barriers to participation during feasibility work because unforeseen challenges with recruitment can and do lead to the early termination of definitive trials [14]. Despite this, a recent systematic review found that only 8% of published pilot and feasibility studies provided detailed coverage of findings related to recruitment [42]. Within our feasibility study, we used brief interviews with non-participants to identify barriers to recruitment. Brief interviews were chosen over a structured questionnaire or open-text boxes to gain insight into and explore the reasons behind non-participation. Although the data collected via these brief telephone interviews were not as rich as with in-depth interviews, the use of brief, structured interviews allowed a greater number of non-respondents to be contacted and the data to be analyzed within the time and financial constraints of the feasibility study.

These interviews identified that the most frequent reason for non-participation were views and perceptions of the pain management course. These findings are in line with previous research, which identified that perceptions of the course and satisfaction with current self-management were reasons for non-participation in a trial of an arthritis self-management program [15]. Difficulty in getting to the hospital was the second most frequent reason for non-participation, despite the offer of reimbursement of travel costs or a pre-paid taxi. Travel issues and the burden of additional appointments are commonly reported barriers to trial participation [15,43]. Future trials of group-based interventions may benefit from consideration of the location of the intervention. For example, interventions held in the community may have greater uptake than those delivered in a hospital, although trials of community-based group interventions also found that difficulties with travel is a common reason for non-participation [15]. Conducting these short interviews with non-participants identified a number of barriers to participation that could be addressed in further refinement work, highlighting the importance and value of conducting research with non-participants in feasibility studies. Based on our findings, we would advocate that brief interviews with non-participants should form a core component of pilot and feasibility studies.

### Recruitment, retention and outcomes assessment

The recruitment rate for this trial was 23%, which is lower than the 42% to 79% recruitment rates reported in previous trials of pain self-management interventions for patients undergoing joint replacement [11,12]. However, other feasibility and pilot studies using a postal recruitment method have reported similarly low response rates [25,44,45]. Despite the low recruitment rate, retention of participants and questionnaire completion were high and similar between the trial arms, suggesting that randomization and outcomes assessment were acceptable.

Recruitment into trials is known to be challenging and considerable research has been conducted into improving trial recruitment. Methods such as telephone reminders to non-responders, ‘opt-out’ recruitment strategies and financial incentives have been found to improve recruitment rates [46]. However, potential issues around coercion and undue influence can pose challenges to the implementation of these strategies. Financial incentives for research participation is a debated issue, and ambiguities remain around what level of incentive constitutes undue influence, with little standardized guidance for ethics committees [47]. For example, based on feedback from our patient and public involvement group, we planned to offer participants free one-year membership to Arthritis Care, but the ethics committee perceived this as potentially coercive and asked for this offer to be removed from the study protocol. This demonstrates the challenges researchers can face in implementing measures to maximize recruitment into trials while remaining in keeping with preferences of the NHS research ethics committee.

### Economic evaluation

Economic evaluations within clinical trials are prone to missing data and therefore we explored whether it was feasible to collect resource-use data using self-complete questionnaires [48]. The economic evaluation work highlighted the difficulty of collecting resource-use data from randomization until surgery for this patient group. However, average waiting time for surgery in this patient group was three months, and we would not expect the intervention to lead to behavior change that would produce differences in cost drivers in the shorter term. In comparison to the pre-operative diaries, the post-operative resource-use questionnaires achieved good completion rates, allowing for a health and social care payer evaluation perspective to be taken. The completion rates could be further improved after imputation of community-based resources data. Although completion rates for a societal perspective were low, categories on productivity losses and informal carer time were well-completed and can be of added value to a sensitivity analysis in a definite economic evaluation.

### Acceptability of the intervention

In addition to assessing trial processes, this study evaluated the acceptability of the intervention. Feedback on the course was positive, suggesting that the course was acceptable and well-received by those who attended. In particular, positive feedback was received on the group-based format, with patients commenting that they appreciated the opportunity to meet other people undergoing THR surgery. Studies evaluating group-based interventions in other clinical settings have also reported positive feedback on this format of intervention delivery [27,44,49]. Therefore, although the group format was a reason for non-participation for some patients, those who attended the course enjoyed the format and engagement with other patients. This highlights an issue affecting many trials: a potentially bias sample because of the self-selection of participants with a preference for the intervention. Differences in the characteristics of participants and non-participants is well known, with an under-representation of older people, women and ethnic minorities in clinical research [50]. Addressing willingness to participate due to the nature of the intervention in feasibility work has the potential to lead to refinements in the intervention for a definitive trial, and this knowledge has implications for the roll-out and uptake of interventions if subsequently implemented in clinical practice.

The Challenging Pain and Keep Challenging Pain courses were highly rated by participants, however attendance at the post-operative course was lower than the pre-operative course. Reasons given for non-attendance were predominantly because people were unavailable on the dates set for the course. The logistics of scheduling group-based interventions is challenging, as many patients have limited availability due to other commitments [15,27]. Increasing flexibility in the scheduling of group-based interventions can be challenging, particularly within the financial constraints of a trial, but having the flexibility to run multiple courses is an important factor to consider when costing a trial. Our short interviews with non-participants also highlighted the importance of offering courses outside of working hours to avoid disadvantaging those patients in employment from participating in clinical trials.

## Conclusions

Undertaking feasibility work for an RCT and evaluating the acceptability of an intervention can be a labor-intensive exercise. However, this study highlights the importance of conducting such work prior to undertaking a full-scale RCT to assess the effectiveness and cost-effectiveness of an intervention. In particular, interviews with non-participants provided valuable information about barriers to participation. The low recruitment rate and poor attendance at the intervention suggest that roll out of the feasibility study to a definitive trial in its current design at our center would not be feasible. Further research would be necessary to evaluate strategies to improve recruitment rates and increase flexibility in the scheduling of the group-based intervention. However, questionnaire completion rates, retention of participants and satisfaction ratings with the intervention were all high, suggesting that further methodological work could lead to a feasible trial design.

Although this study was limited to a single orthopedic center, several key messages can be taken from our experience. First, conducting brief telephone interviews with non-participants is an efficient method of collecting data on barriers to participation, and we recommend it should be a core component of feasibility studies. These data can also provide insight into whether unwillingness to participate is due to the nature of the intervention, thereby providing early indications of potential issues in a definitive trial and with uptake of the intervention if implemented into clinical practice. Second, attempts to implement methods to improve patient recruitment need to be carefully designed in light of ethical considerations, such as the potential for inducements to be seen as coercion. Third, the logistical difficulties in scheduling groups and ensuring high attendance should not be underestimated and the potential to increase flexibility by running multiple courses should be considered when designing a budget for a trial. Fourth, the ability of piloting resource-use questionnaires is a major advantage to improve the quality of resource-use data available in the definitive economic evaluation. Finally, given the need to ensure that research is efficient and provides value for money, our study highlights that feasibility studies are able to identify areas that should be considered in the design or commissioning of research addressing similar interventions or populations.

## Abbreviations

CI: confidence interval; IQR: interquartile range; NHS: National Health Service; PSS: Personal Social Services; RCT: randomized controlled trial; SD: standard deviation; THR: total hip replacement; WOMAC: Western Ontario and McMaster Universities Osteoarthritis Index.

## Competing interests

The authors declare that they have no competing interests.

## Authors’ contributions

VW, EM, NA, AB and RGH designed the study. VW coordinated the study and was responsible for the acquisition of data. VW, EM and RGH analyzed and interpreted the data. VW drafted the manuscript and EM, NA, AB and RGH revised the manuscript critically for important intellectual content. All authors read and approved the final manuscript.

## Supplementary Material

Additional file 1 CONSORT 2010 Flow Diagram. Click here for file
