# Supplementary material for: Effectiveness and cost-effectiveness of a group-based pain self-management intervention for patients undergoing total hip replacement: feasibility study for a randomized controlled trial
Source: Trials. 2014 May 20;15:176. doi: 10.1186/1745-6215-15-176 (PMC4031159; doi:10.1186/1745-6215-15-176)
Supplement: Additional file 1 — CONSORT 2010 Flow Diagram. [file 1745-6215-15-176-S1.doc]

**CONSORT 2010 Flow Diagram**

**Allocation**

**Analysis**

**Follow-Up**

**Enrollment**

Assessed for eligibility (n=409)

Excluded (n=321)

♦  Not meeting inclusion criteria (n=24)

♦  Declined to participate (n=297)

♦

Number of patients analysed: not applicable – feasibility study

Lost to follow-up (self-withdrawal, recruited into another trial, did not have surgery) (n=7)

Allocated to intervention (n=43)

♦ Received allocated intervention (n=28)

♦ Did not receive allocated intervention (n=15)

Lost to follow-up (Self-withdrawal, did not have surgery) (n= 8)

Allocated to usual care (n=45)

♦ Received allocated intervention (n=45)

♦ Did not receive allocated intervention (n=0)

Number of patients analysed: not applicable – feasibility study

Randomized (n=88)
